# Re-irradiation for isolated neck recurrence in head and neck tumor: impact of rN category

**DOI:** 10.1038/s41598-024-53438-w

**Published:** 2024-02-07

**Authors:** Hideya Yamazaki, Gen Suzuki, Norihiro Aibe, Hiroya Shiomi, Ryoong-jin Oh, Ken Yoshida, Satoaki Nakamura, Koji Konishi, Tomohiko Matsuyama, Mikio Ogita

**Affiliations:** 1 Department of Radiology, Graduate School of Medical Science, Kyoto Prefectural University of Medicine, 465 Kajiicho Kawaramachi Hirokoji, Kamigyo-Ku, Kyoto 602-8566 Japan; 2 CyberKnife Center, Soseikai General Hospital, Kyoto, Japan; 3 Department of Radiation Oncology, Miyakojima IGRT Clinic, Osaka, Japan; 4 Department of Radiology, Kansai Medical University, Hirakata, Japan; 5 Department of Radiation Oncology, Osaka International Cancer Institute, Osaka, Japan; 6 Department of Radiation Oncology, Kumamoto University Hospital, Kumamoto, Japan; 7 Radiotherapy Department, Fujimoto Hayasuzu Hospital, Miyakonojo, Japan

**Keywords:** Head and neck cancer, Re-irradiation, Stereotactic radiotherapy, Oral cancer, Pharyngeal cancer, Laryngeal cancer, Cervical lymph node, Isolated recurrence, Cancer, Medical research, Oncology

## Abstract

Unresectable, isolated lymph node recurrence after radiotherapy is rare but a candidate for re-irradiation. However, severe toxicity is anticipated. Therefore, this study aimed to explore the efficacy and toxicity of re-irradiation in isolated lymph node recurrence of head and neck lesions. We analyzed 46 patients who received re-irradiation for lymph node recurrence without local progression. The primary tumor sites included the oral cavity in 17 patients, the hypopharynx in 12, the oropharynx in seven, the larynx in three, the nasopharynx in two, and other sites. During a median follow-up time of 10 months, the median survival time was 10.6 months, and the 1-year overall survival rate was 45.5%. The 1-year local control and progression-free survival rates were 49.8% and 39.3%, respectively. According to univariate analysis, age (≥ 65 years), the interval between treatment (≥ 12 months), rN category (rN1), and gross tumor volume (GTV < 25 cm^3^) were predisposing factors for better survival. In the multivariate analysis, the rN category and interval were identified as statistically significant predictors. Late toxicity grade ≥ 3 occurred in four patients (8.6%). These were all Grade 5 carotid blowout syndrome, which associated with tumor invasion of the carotid artery and/ or high doses administration for the carotid artery. Small-volume rN1 tumor that recur after a longer interval is a feasible candidate for re-irradiation. However, strict patient selection and meticulous care for the carotid are required.

## Introduction

The major pattern of failure after the definitive treatment of head and neck cancers with chemoradiotherapy with/without surgery continues to be locoregional failure within high-dose radiotherapy^1–3^. Although isolated neck recurrence without local recurrence or distant metastasis is uncommon, it occurs in 5–10% of patients after radical treatment^4–6^. Salvage surgery is the best curative treatment to prolong survival. However, it is difficult in almost all cases and is feasible only in 7–27% of patients because of extensive tumor invasion and a high risk of postoperative complications (e.g., wound healing problems and fistulas) in the irradiated area^6–9^. Systemic therapy is the next choice of treatment for neck cancer recurrence after chemoradiotherapy^10–12^. However, the median survival time (MST) after systemic chemotherapy is reportedly 5–13 months^9–13^. Moreover, persistent isolated lymph nodes are common, and lymph node metastases represent the entire disease burden in patients who cannot undergo surgery or systemic therapy. In such cases, local therapy could be an effective treatment option for alleviating symptom palliation and improving oncologic outcomes^14–26^. Re-irradiation has been explored as an optional local therapy for unresectable head and neck cancers. The installation of advanced techniques (stereotactic body radiotherapy (SBRT) and/or intensity-modulated radiotherapy (IMRT) enabled us to irradiate the target volume without unnecessary higher irradiation to adjacent normal tissue. However, a heterogeneous patient population makes it challenging to interpret the role of re-irradiation^14–31^. Additionally, severe toxicity is anticipated after re-irradiation of the head and neck lesions^14–31^. Of these, lymph node re-irradiation, excluding Rouviere node, showed a relatively higher carotid blowout syndrome (CBOS) rate (11/90 = 12.2%) than other sites (21/291 = 7.2%)^31^. This study aimed to investigate re-irradiation efficacy and toxicity in patients with limited lymph node metastases using a multi-institutional database.

## Materials and methods

### Patients

We analyzed patients with isolated (without local recurrence) recurrent cervical lymph node metastases from head and neck lesions treated at seven institutions between 2002 and 2018. The inclusion criteria were as follows: (i) Re-irradiation in the same area as the previous radiotherapy, performed 30 Gy in 10 fractions or more (equivalent 2-Gy fractions = EQD2 ≥ 36 Gy, using α/β = 3 Gy), (ii) Histology confirming the pathology before the initial treatment, (iii) Recurrence after curative-intent treatment, including chemotherapy, surgery, and radiotherapy, (iv) No primary tumor progression, (v) Eastern Cooperative Oncology Group performance status scores of 0–2, (vi) Inoperable status according to the opinions of the head and neck surgeons; unresectable or medically inoperable because of severe coexisting disease or something else.

The exclusion criteria were as follows: (i) distant metastasis, (ii) palliative radiotherapy for symptomatic relief (e.g., 6–8 Gy/1 fraction), and (iii) planned boost radiotherapy and/or clinically residual cases (progression case at completion of initial radiotherapy). The study was conducted in accordance with the guidelines of the Declaration of Helsinki and approved by the Institutional Review Board of Kyoto Prefectural University of Medicine (ERB-C-1330–3).

Three patients underwent conventional three dimensional conformal radiation therapy (3D-CRT), eight underwent intensity modulated radiotherapy (IMRT), and 35 underwent stereotactic body radiotherapy (SBRT). The gross tumor volume (GTV) was defined as visible tumor on CT/MRI images. The planning target volume (PTV) is determined by adding an adequate margin to the GTV. For example, GTV = CTV = PTV in several institutions with CyberKnife, and GTV = CTV and PTV = CTV + 2–5 mm in several institutions with LINAC. A dose of 32 Gy (median, range, 12–60 Gy) in 5 fractions (median, range, 1–30 fractions) (Table 1) was prescribed. Equivalent Dose in 2 Gy fraction (EQD2) was estimated according to the following equation: EQD2 = n × d × ((α/β) + d)/((α/β) + 2), where n is the number of treatment fractions, d is the dose per fraction in Gy, and α/β = 10 Gy for tumor and α/β = 3 Gy for normal tissue toxicity.

**Table 1:** Patients characteristics.

Variables	Group	Median or PT NO (n = 46)	(%) or range
Age		64	33–87
Gender	Female	11	(23.9%)
	Male	35	(76.1%)
Primary site	NPC	2	(4.3%)
	OPC	7	(15.2%)
	HPC	12	(26.1%)
	Oral	17	(37.0%)
	Laryngeal	3	(6.5%)
	Others	5	(10.9%)
rN category	1	16	(34.8%)
	2(2a:2b:2c)	14(6:6:2)	(30.4%)
	3	16	(34.8%)
Histology	SCC	43	(93.5%)
	Other	3	(6.5%)
Location	Rouviere	8	(17.4%)
	Other	38	(82.6%)
Previous surgery	No	15	(32.6%)
	Yes	31	(67.4%)
Chemotherapy	No	35	(76.1%)
	Yes	11	(23.9%)
Goss tumor volume (GTV)	cm^3^	14.1	1.6–113
Prescribed dose	(Gy)	32	12–60
Fractionation	(fractions)	5	1–30
EQD2Gy	(Gy)	43.7	23.3–84.2
Interval between treatment	(months)	11.5	1–374
Previous prescribed dose	(Gy)	60	23–72
Previous fractionation	(fractions)	30	2–60
Follow-up	(months)	10.6	4–84

EQD2Gy = n × d ([α/β] + d)/([α/β] + 2).

*n* Number of treatment fractions; *d* Dose. Oher histology included sclerosing ductal ca, papillary ca. mucoepidermoid ca. *NPC* Nasopharyngeal ca., *OPC* Oropharyngeal ca., *HPC* Hypopharyngeal ca., Others primary sites included thyroid, external ear, left Auricle, skin, and maxillary sinus. *SCC* Squamous cell carcinoma.

The primary endpoint was overall survival (OS). The secondary endpoints were local control (LC), progression-free survival (PFS), and toxicity grade ≥ 3. Survival data were calculated from the start of re-irradiation based on the first-event analysis for all endpoints. For these analyses, LC was defined as disease progression in the treated lymph nodes or death. PFS was defined as disease progression, relapse, or death from any cause. Toxicity was determined using the Common Terminology Criteria for Adverse Events Version 4.0. CT/MRI/PET images were used to determine the tumor stage according to the Union for International Cancer Control (UICC) TNM Classification of Malignant Tumors, version 7.

### Statistical analysis

Actuarial statistics for clinical outcomes were calculated using StatView 5.0, statistical software (SAS Institute, Cary, NC, USA) and EZR-stat^32^. A chi-square test was used to analyze the frequency. Means were compared using the Mann–Whitney U test for skewed data and the student’s t-test for normally distributed data. Survival data were calculated using the Kaplan–Meier method and examined for significance using a log-rank test. Univariate and multivariate analyses of local control and survival rates were performed using Cox proportional hazards models. We examined the following factors in the univariate analysis: age, sex, location, histology, previous surgery, chemotherapy, gross tumor volume, interval between treatments, and prescribed dose. Statistically significant variables in the univariate analysis were included in the multivariate analysis. The primary tumor location (nasopharyngeal cancer or not) was omitted from this analysis because of the small number of patients with nasopharyngeal cancer (n = 2). Cutoff values were set at each variable’s median value or average unless otherwise stated: 25 cm^3^ for the GTV, which was set in a previous analysis^33^. Statistical significance was set at *p *< 0.05.

### Informed consent

Informed consent was obtained from all subjects involved in the study.

## Results

Table 1 summarizes the patient, tumor, and treatment characteristics.

**Figure 1:**
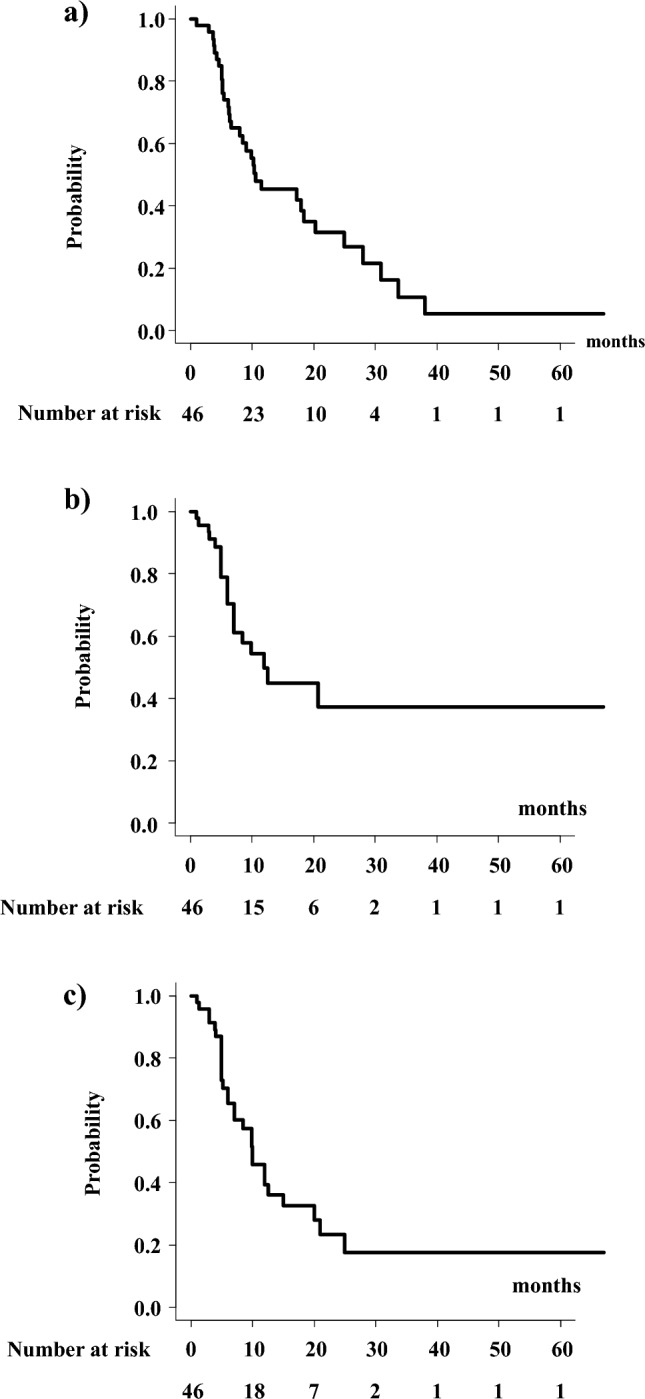
(**a**) Overall survival rate. (**b**) Local control rate. (**c**) Progression-free survival rate.

With a median follow-up period of 10.6 months (range, 4–84 months), the median survival time was 10.8 months (95% confidence interval: 95% CI 6.6–20.3 months), with a 1-year survival rate of 45.5% (95% CI 30.2–59.5%, Fig. 1a). Among 46 patients, 20 experienced local failure during the follow-up period, and the 1-year local control rates were 49.8% (95% CI 31.7–65.6%, Fig. 1b). Progression-free survival was 39.3% (95% CI 23.7–54.6%) at 1 year. The median value was 10 months (95% CI 6–5 months, Fig. 1c).

The results of the analysis of the predisposing factors for OS are shown in Table 2. Younger age of ≤ 65 years, larger tumor size of > 25 cm^3^, advanced N category (rN2-3), and short interval between initial radiotherapy and re-irradiation ≤ 12 months were statistically significant predisposing factors for poor OS in univariate analysis (Table 2). Of these, we found that the rN category and interval were statistically significant predisposing factors for OS in multivariate analysis.

**Table 2:** Uni- and multivariate analysis for overall survival rate using Cox proportional hazards model.

Variable	Strata	Univariate	Multivariate analysis	
		*P value*	Hazard ratio (95% CI)	*P value*
Age, years	< 65 versus 65 ≤	**0.02182**	0.6462 (0.2614–1.5970)	0.34440
Gender	Female versus Male	0.634		
Location	Rouviere versus other	0.2333		
Histology	scc versus not scc	0.6155		
N category	1 versus 2–3	**0.001197**	5.3310 (1.5680–18.12)	**0.00736**
Previous surgery	No versus Yes	0.7173		
Chemotherapy	No versus Yes	0.2475		
Goss tumor volume (GTV)	≤ 25 cm^3^ versus 25 cm^3^ <	**0.01419**	0.9303 (0.3742 -2.313)	0.8764
Interval between treatment	≤ 12 months versus 12 months <	**0.04283**	0.3373 (0.1395–0.8158)	**0.01587**
Prescribed dose	EQD2 ≤ 40 Gy versus EQD2 > 40 Gy	0.7514		

Bold values indicate statistically significance.

*CI* Confidence interval, *OS* Overall survival, *scc* Squamous cell carcinoma.

**Figure 2:**
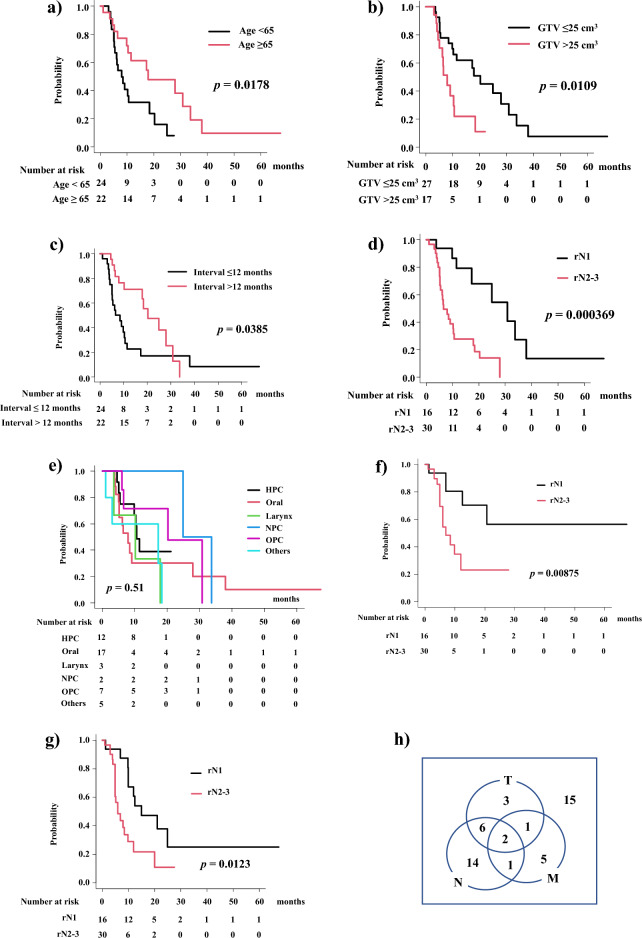
(**a**) Overall survival rate according to age. (**b**) Overall survival rate according to GTV. (**c**) Overall survival rate ac-cording to the interval. (**d**) Overall survival rate according to rN category. (**e**) Overall survival rate according to primary sites. HPC = hypopharyngeal ca. NPC = nasopharyngeal ca., OPC = oropharyngeal ca. (**f**) Local control rate according to rN category. (**g**) Progression free survival rate according to rN category. (**h**) Failure pattern. T = local failure, N = nodal failure, M = distant metastases.

Older patients aged ≥ 65 years, showed superior 1-year OS of 61.1%, compared with younger patients aged < 65 years, with OS of 31.6% (Fig. 2a, *p* = 0.0178). Analyzing by GTV, patients with smaller GTV (≤ 25 cm^3^) showed superior 1-year OS (61.9%) than the larger GTV group (22.0%, Fig. 2b, *p* = 0.0109). Analyzing the interval between previous radiotherapy and re-irradiation, patients with a longer interval (12 months ≤) showed superior 1-year OS of 71.2% than those with a shorter interval (< 12 months) (22.7%, Fig. 2c, *p* = 0.0385). Patients with rN1 disease showed superior 1-year OS of 79.3% compared with patients with rN2-3 diseases who had OS of 27.7% (Fig. 2d, *p* = 0.000369; 1-year OS of 42.9% for rN2 and 13.8% for rN3). For primary sites, 1-year OS were 38.9%, 30.3%, 33.3%, 100%, 71.4%, and 60% for hypopharyngeal cancer, oral cancer, laryngeal cancer, nasopharyngeal cancer, oropharyngeal cancer, and others (Fig. 2e, *p* = 0.51), respectively. Patients with rN1 disease showed superior 1-year LC rates of 80.4% compared with patients with rN2-3 diseases whose LC rates were 23.0% at 1 year (Fig. 2f, *p* = 0.00875; hazard ratio 3.722, *p *= 0.01511; Supplemental Table S1). Patients with rN1 disease showed superior 1-year PFS of 60.6% compared with patients with rN2-3 diseases with PFS of 21.8% (Fig. 2g, *p* = 0.0123; hazard ratio 2.616, 95% CI = 1.182–5.79, *p *< 0.0177; Supplemental Table S2).

Nine patients (19%) experienced distant metastases (seven to the lungs, one to the skin, and one to the mediastinal lymph node), resulting in death. Figure 2h shows the failure patterns of the diseases.

### Toxicity

Four grade 2 toxicities (oral mucositis, pain with dermatitis, pain, and ulceration) and three Grade 1 toxicities (dermatitis, swallowing pain, and alopecia) appeared after re-irradiation (Table 3).

**Table 3:** Patients and treatment characteristics of four cases with carotid blow-out syndrome (CBOS).

PTNO	Age/gender	Primary site	Histology	Previous surgery	Previous chemotherapy	Previous RT	GTV (cm^3^)	Encasement of carotid more than > 180˚	ReRT	Total EQD2Gy (a/b = 3)	Time to CBOS (months)
1	66 F	HPC	SCC	Yes	No	60 Gy/ 30 fr	32.2	Yes	27 Gy /5fr	105.4	4.6
2	63 M	OPC	SCC	Yes	No	60 Gy/30 fr	22.0	Yes	30 Gy /5fr	114	27.5
3	37 M	Oral	SCC	Yes	Yes	62 Gy/31 fr	32.5	Yes	60 Gy/30fr	122	6.2
4	33 F	Oral	SCC	Yes	Yes	50 Gy/25 fr	5.6	No	50 Gy/10fr	130	8.5

Four cases showed late toxicity grade ≥ 3; all had lethal carotid blow-out syndrome (CBOS) Grade 5. Three cases showed both encasement of the carotid > 180° by tumor involvement (GTV = 32.29 cm^3^, 22.09 cm^3^, and 32.5 cm^3^) and post-operative status (treatment intervals 61, 24, and 4 months). The prescribed doses for initial radiotherapy were 60 Gy/30 fractions, 60 Gy/30 fractions, and 62 Gy/31 fractions, respectively. For re-irradiation, 27 Gy/5 fractions, 30 Gy/5 fractions, and 60 Gy/30 fractions were used. Therefore, the summation of EQD 2 was 105.4, 114, and 122 Gy (α/β = 3), respectively. Another case was a 33-year-old female with tongue cancer isolated neck recurrence (GTV = 5.6 cm^3^) without encasement of the carotid more than > 180° by tumor involvement and post-operative status (treatment intervals of 2 months). The prescribed dose for initial chemoradiotherapy was 60 Gy/30 fractions. Re-irradiation dose of 50 Gy/10 fractions (cumulative dose was 130 Gy in EQD2) using the IMRT technique with TS-1 six months later for lymph node recurrence (the right upper deep jugular chain). She showed bleeding 8.5 months after reirradiation.

## Discussion

This study aimed to examine the re-irradiation efficacy and toxicity of isolated neck lymph node recurrences in head and neck tumors after curative radiotherapy using multi-institution data. To the best of our knowledge, this is one of the largest series of re-irradiation cohorts for rare, isolated neck lymph node recurrences. Furthermore, this is the first study to demonstrate the importance of the earlier rN category (rN1) for re-irradiation.

Advancements in radiotherapy techniques have brought re-irradiation into focus for unresectable and systemic therapy infeasible recurrent head and neck cancers. Numerous studies have explored the role of re-irradiation in head and neck cancers, including research conducted in our department^14–31,34,35^. 3D-CRT and advanced delivery techniques such as IMRT, SBRT have been explored to deliver high doses to tumors while minimizing the radiation dose to surrounding normal structures, but data on its efficacy and safety for recurrent head and neck cancer are limited. SBRT used a unique target volume definition (GTV = CTV = PTV) in several Cyberknife institutions for precise dose delivery system. In 3D-CRT, generally, the isocenter was chosen as the dose prescribed point. In SBRT and IMRT, the volume dose prescription was used. For instance, D95 (dose encompassing 95% of the volume of PTV) was used for SBRT or IMRT. For dose distribution, IMRT could create dose distributions tailored to tumor morphology and SBRT could create steep dose gradient.

Strojan et al. reported two-year overall survival rates ranging from 10 to 30% after re-irradiation (mainly local recurrences), Grade 3–4 late effects as common as 40%, and 10% of Grade 5 due to carotid blow-out syndrome, hemorrhage, sepsis, etc. detected^28^. The result of IMRT was based on observational studies demonstrating 2-year overall survival (46%, 95% CI = 41%-50%)^29^. The pooled rates of late grade ≥ 3 and grade 5 toxicities were 26% (95% CI = 20%-32%) and 3.1% (95% CI = 2%-5%), respectively. The pooled 2-year OS following SBRT was 30.0% (95% CI = 24.5–36.1%) The pooled rates of late grade ≥ 3 and grade 5 toxicities were 9.6% (95% CI = 5.0–17.6%) and 4.6% (95% CI = 2.4–8.6%), respectively^30^. Investigations are ongoing to further evaluate the integration of newer radiation techniques for re-irradiation. Most published studies have included heterogeneous populations, mainly those with local recurrences at many primary sites. Recurrence of isolated neck lymph nodes is rare^4–6^. However, the biological characteristics of neck lymph node recurrences require further consideration.

For re-irradiation of lymph nodes, Kawaguchi et al. reported that among the eight patients with lymph node metastases in their study, one patient with a single retropharyngeal (12.5%) had a complete response; the remaining seven patients (87.5%) all progressed^34^. Kobayashi et al. reported that the 2-year LC and OS were 81.4% and 46.3%, respectively^26^. LC was higher with a target volume ≤ 1.0 cm^3^ than that with a target volume > 1.0 cm^3^ (*p *= 0.006). Fatal bleeding was observed in one patient with a widespread tumor that invaded the carotid artery. Pollard et al. also reported good outcomes from re-irradiation for small retropharyngeal nodal metastases with a high prescribed dose without Grade ≥ 3 late toxicity^27^. Among 19 patients, the 1-year LC, locoregional control, OS, and PFS were 100%, 94%, 92%, and 92%, respectively. For entire head neck cancer re-irradiation, Diao et al. reported 44.3 months of MST with a 1-year local control rate of 78% using 45 Gy/ 9 fractions of SBRT for a median target volume of 16.9 cm^3^ (small tumors) in 137 patients^34^. Our data concur with these findings and suggest that smaller lesions are associated with better outcomes. This is reflected in the traditional Union for International Cancer Control T category, which uses the diameter of oral, oropharyngeal, and hypopharyngeal cancers to classify a tumor into either T1 (-2 cm) or T2 (-4 cm). This reflects 4.1 cm^3^ and 33.4 cm^3^ in spherical tumor volume. In addition, we found that rN1 (ipsilateral single lymph node ≤ 3 cm) and interval were important predictors of prognosis, which concurred with the discussion for the tumor volume factor. The importance of tumor volume has been highlighted in the literature^12,21–23,28–30,34,35^. Small-volume isolated rN1 cervical lymph node-oriented directed therapy, including re-irradiation, may play a role in some cases. We prescribe 60 Gy or more in EQD2G for small lesions (i. e., rN1) according to recent studies.

The treatment interval is also an important predisposing factor for re-irradiation in the literature^12,21–23,28–30,34,35^. Age is sometimes reported to be an important predisposing factor for radiotherapy^36^; however, this has yet to be confirmed^35^. We speculate that younger patients showed more advanced disease characteristics than older patients (i.e., rN2-3 tumor, shorter intervals with borderline significance) (Supplemental Table S3). Thus, we believe that age does not result in a poorer prognosis in the absence of other risk factors.

We encountered four cases of CBOS, which is one of the most devastating complications of head and neck cancer treatment and mainly occurs in patients with a history of radiotherapy for tumors involving the vascular axis^31,37–46^. Grimm et al. reported that risk factors for CBOS include a greater degree of circumferential tumor involvement around the major vessel, consecutive daily treatments, and surgical procedures before or after radiotherapy^38^. Additionally, the presence of ulceration, skin invasion, necrosis/infection, and irradiation of the lymph node area adjacent to the carotid may be risk factors for CBOS^31,38–48^. McDonald et al. reported that CBOS after re-irradiation is a rare [41/1,554 (2.6%)] and often fatal (75%) event^43^. We also reported that CBOS occurred in 8.4% of cases among 381 patients treated with 484 re-irradiation sessions using stereotactic radiotherapy, and 69% of these cases were fatal^31^. Embring et al. proposed a maximal accumulated EQD2 (previous radiotherapy plus re-irradiation) of 120 Gy as a dose constraint for CBOS^40^. The Turkish group reported that CBOS was not found in cases with less than 180° of carotid invasion or D0.1cc < 47.6 Gy/ 5 fractions on nonconsecutive days^41,42^. In our study, three out of four cases showed encasement of the carotid > 180°, and all cases had postoperative recurrence with the cumulative prescribed dose of EQD2 > 105 Gy. The median total summation of EQD2 for patients with CBOS was 118 Gy (range 105.36–130 Gy), whereas it was 114 Gy (78.00–145.15) for patients without CBOS (*p *= 0.654). Our data are in line with those of Thariat et al.^44^, who suggested maintaining the maximum cumulative dose to the carotid artery below EQD2 100 Gy in patients treated with SBRT.

Recent advances have emphasized the use of immunotherapy in difficult clinical cases. In 2019, the FDA approved pembrolizumab (a PD-1 inhibitor) as a first-line treatment for patients with metastatic or unresectable recurrent head and neck squamous cell carcinoma^47^. Combining this with re-irradiation could improve effectiveness, as radiotherapy can modulate the immune system and improve immunotherapy^48^. Prior studies have demonstrated improved local control with conventional re-irradiation combined with systemic therapy^15–17^.

This study had several limitations. First, it included small sample size, heterogeneous tumor subsites and histologies, and a limited follow-up time. Moreover, heterogeneous tumor locations and histological findings prevented discrete conclusions and increased selection bias risk. However, our study is one of the largest on re-irradiation for isolated cervical lymph node recurrence and reveals the importance of small-volume rN1 that recurred after a longer interval; therefore, the findings may be useful.

## Conclusions

Although strict patient selection and meticulous care for the carotid are required (8.6% of deaths due to CBOS), small-volume rN1-isolated lymph node recurrence in head and neck lesions that recur after a longer interval may be a candidate for re-irradiation. A multidisciplinary approach for determining the optimal dose and irradiation schedule should be prospectively evaluated.

### Supplementary Information

Supplementary Tables.

## Data Availability

The datasets used and/or analysed during the current study are available from the corresponding author on reasonable request.
