# Supplementary material for: Re-irradiation for isolated neck recurrence in head and neck tumor: impact of rN category
Source: Sci Rep. 2024 Feb 7;14:3107. doi: 10.1038/s41598-024-53438-w (PMC10850055; doi:10.1038/s41598-024-53438-w)
Supplement: Supplementary file 1 — Supplementary Tables. [file 41598_2024_53438_MOESM1_ESM.docx]

**Supplemental Table S1.** Univariate analysis for local control rate using Cox proportional hazards model.

| Variable | Strata |  | |
| --- | --- | --- | --- |
|  |  | Hazard ratio  (95% CI) | *P-value* |
| Age, years | < 65 vs 65 ≤ |  | 0.3291 |
| Gender | Female vs Male |  | 0.2007 |
| Location | Rouviere vs other |  | 0.2223 |
| Histology | scc vs not scc |  | 0.9994 |
| N category | 1 vs 2-3 | 3.722 (1.289-10.75) | **0.01511** |
| Previous Surgery | No vs Yes |  | 0.0604 |
| Goss tumor volume (GTV) | ≤ 25 cm^3^ vs 25 cm^3^ < |  | 0.2826 |
| Interval between treatment | ≤ 12 months  vs 12 months < |  | 0.3124 |
| Prescribed dose | EQD2 ≤ 40Gy  vs EQD2 > 40Gy |  | 0.6326 |

Bold values indicate statistical significance. CI = confidence interval, scc = squamous cell carcinoma.

**Supplemental Table S2.** Univariate analysis for progression free survival rate using Cox proportional hazards model.

| Variable | Strata |  | |
| --- | --- | --- | --- |
|  |  | Hazard ratio  (95% CI) | *P-value* |
| Age, years | < 65 vs 65 ≤ |  | 0.1795 |
| Gender | Female vs Male |  | 0.8537 |
| Location | Rouviere vs other |  | 0.7231 |
| Histology | scc vs not scc |  | 0.9979 |
| N category | 1 vs 2-3 | 2.616 (1.182-5.79) | **0.0177** |
| Previous Surgery | No vs Yes |  | 0.09439 |
| Goss tumor volume (GTV) | ≤ 25 cm^3^ vs 25 cm^3^ < |  | 0.1899 |
| Interval between treatment | ≤ 12 months  vs 12 months < |  | 0.1293 |
| Prescribed dose | EQD2 ≤ 40Gy  vs EQD2 > 40Gy |  | 0.9889 |

Bold values indicate statistical significance. CI = confidence interval, scc = squamous cell carcinoma

**Supplemental table S3. Patients’ characteristics according to age.**

| Factor | Group | Age < 65 | Age 65 ≤ | *p* - value |
| --- | --- | --- | --- | --- |
|  |  | n = 24 | n = 22 |  |
| Age |  | 56.50 [33.00, 64.00] | 75.00 [65.00, 87.00] | **<0.001** |
| Gender | Female | 6 (25.0) | 5 (22.7) | 1 |
|  | Male | 18 (75.0) | 17 (77.3) |  |
| Primary site | NPC | 1 (4.2) | 1 (4.5) |  |
|  | OPC | 6 (25.0) | 1 (4.5) | 0.207 |
|  | HPC | 4 (16.7) | 8 (36.4) |  |
|  | Oral | 10 (41.7) | 7 (31.8) |  |
|  | Laryngeal | 1 (4.2) | 2 (9.1) |  |
|  | Others | 2 (8.4) | 3 (13.5) |  |
| Histology | scc | 24 (100.0) | 20 (86.4) | 0.101 |
|  | Other | 0 (0.0) | 2 (13.6) |  |
| Location | Rouviere | 1 (4.2) | 7 (31.8) | **0.02** |
|  | Other | 23 (95.8) | 15 (68.2) |  |
| Previous Surgery | No | 6 (25.0) | 9 (40.9) | 0.348 |
|  | Yes | 18 (75.0) | 13 (59.1) |  |
| Goss tumor volume (GTV) | cm^3^ | 19.10 [1.59, 113.00] | 14.00 [3.11, 65.70] | 0.364 |
| rN category | rN1 | 5 (20.8%) | 11 (50.0%) | 0.063 |
|  | rN2-3 | 19 (79.2%) | 11 (50.0%) |  |
| Chemotherapy | No | 18 (75.0) | 17 (77.3) | 0.452 |
|  | Yes | 6 (25.0) | 5 (22.7) |  |
| Prescribed does | (Gy) | 31.00 [18.00, 60.00] | 32.50 [12.00, 60.00] | 0.557 |
| Fractionation | (fractions) | 5.00 [3.00, 30.00] | 5.00 [1.00, 20.00] | 0.831 |
| EQD2 | Gy | 46.85 [23.30, 84.24] | 43.72 [23.33, 70.00] | 0.877 |
| Interval between treatment | (months) | 10.00 [3.40, 184.00] | 14.50 [3.00, 374.00] | 0.508 |
| Previous prescribed does | (Gy) | 60.00 [23.00, 72.00] | 60.00 [25.00, 70.00] | 0.798 |
| Previous fractionation | (fractions) | 30.00 [2.00, 60.00] | 30.00 [2.00, 35.00] | 0.982 |
| Follow-up | (months) | 7.50 [3.00, 27.57] | 11.48 [1.00, 84.00] | 0.075 |

| Bold values indicate statistically significance |
| --- |
| Abbreviations; NPC = nasopharyngeal ca., PPC = oropharyngeal ca., HPC = hypopharyngeal ca., SCC = squamous cell carcinoma |
